# Polyandry may mitigate the negative impact of reproductive interference among bumblebees in Japan

**DOI:** 10.1007/s00114-024-01917-5

**Published:** 2024-05-23

**Authors:** Fumina Inokuchi, Maki N. Inoue, Yuya Kanbe, Masaaki Ito, Jun-ichi Takahashi, Tetsuro Nomura, Koichi Goka, Koji Tsuchida

**Affiliations:** 1 Laboratory of Insect Ecology, Faculty of Applied Biological Sciences, Gifu University, Yanagido 1-1, Gifu, 501-1193 Japan; 2 Department of Agriculture, Tokyo University of Agriculture and Technology, 3-5-8 Saiwai, Fuchu, Tokyo, 183-8509 Japan; 3 Arysta Lifescience Corporation BioSystems, Asia and Life Science Business Group, 418-404 Nishihara, Tsukuba, Ibaraki 305-0832 Japan; 4 Forestry and Forest Products Research Institute, Aomori Prefectural Industrial Technology Research Center, Hiranai-Machi 46-56, Higashi Tsugaru-Gun, Aomori, 039-3321 Japan; 5 Faculty of Life Sciences, Kyoto Sangyo University, Kyoto, Japan; 6 National Institute of Environmental Studies, Onogawa 16-2, Tsukuba, Ibaraki 305-0053 Japan

**Keywords:** Biological invasion, Polyandry, Reproductive interference, Social insects, Pollinator

## Abstract

In social hymenopterans, monandry of the queen is an ancestral trait, and polyandry is a derived trait. Polyandry of the queen is the norm in a limited number of lineages, such as honeybees, leaf-cutting ants, *Pogonomyrmex* ants, and *Vespula* wasps, which presumably provide fitness advantages for the whole colony. The queen of the introduced bumblebee, *Bombus terrestris*, is polyandrous in Japan, whereas it is monandrous in native regions. We hypothesize that polyandry can evolve in a process that avoids the negative impacts of reproductive interference caused by interspecific mating and conducted genetic studies of the invasive species *B. terrestris* and two native subspecies, *Bombus hypocrita sapporoensis* and *Bombus hypocrita hypocrita*, in Japan. Our results revealed that although the native queens of *B. hypocrita hypocrita* allopatric with *B. terrestris* were strictly monandrous, the native queens of *B. hypocrita sapporoensis* sympatric with *B. terrestris* were polyandrous. These results suggested that the queens of native *B. hypocrita sapporoensis* do not experience negative impacts on interspecific mating from the invasive *B. terrestris*. We discuss the possibility that reproductive interference is a driving force in selection for multiple mating through an arms race between sympatric species.

**Supplementary Information:**

The online version contains supplementary material available at 10.1007/s00114-024-01917-5.

## Introduction

Under haplodiploidy, monogamy maximizes relatedness among sisters (but not among brothers) and is a prerequisite for social evolution (Hughes et al. 2008). In contrast, polygyny and polyandry are considered derived traits that supply genetic variation within a colony and provide several overall fitness advantages for colonies (Boomsma and Ratnieks 1996; Schmid-Hempel and Crozier 1999; Crozier and Fjerdingstad 2001; Strassmann 2001). Although monogamy and high relatedness between donors and recipients are ideal conditions for kin selection, the cause of polyandry or polygyny maintenance in some eusocial lineages has been a central challenge in sociobiology (Crozier and Pamilo 1996). Indeed, the number of queen mates is positively associated with colony reproductive success in *Apis* honeybees (Mattila and Seeley 2007), leaf-cutting ants (Fjerdingstad and Boomsma 1998; Hughes and Boomsma 2004), *Pogonomyrmex* ants (Wiernasz et al. 2004), and *Vespula* wasps (Goodisman et al. 2007; Saga et al. 2020). However, polyandry is also believed to impose several fitness costs on queens, and polygyny likely causes competition among queens over reproduction within a colony.

In bumblebees, only fertilized queens overwinter and establish colonies in spring. Following months in which colonies solely comprise a queen and her offspring workers, the colonies produce new queens and males for reproduction in late summer. After mating, spermatozoa are stored in the spermatheca of the queen until spring when she starts a new nest. Within their native range, *Pyrobombus* species exhibit slight polyandry, whereas *Bombus* species, including *Bombus terrestris*, are monandrous (Estoup et al. 1995; Schmid-Hempel and Schmid-Hempel 2000; Cnaani et al. 2002; Payne et al. 2003; Takahashi et al. 2008a,b; Kokuvo et al. 2009; Huth-Schwarz et al. 2011).

Japan has at least 22 species of native bumblebees; since its deliberate introduction for pollination in 1991, *B. terrestris* has become naturalized (Matsumura et al. 2004; Inoue et al. 2008, 2009). The unrestricted release until 2004 has resulted in noticeable adverse impacts on native fauna and flora, particularly in Hokkaido, the northernmost island of Japan (Dohzono et al. 2008; Tsuchida et al. 2010). Among these impacts, reproductive interference between invasive *B. terrestris* and native *Bombus hypocrita sapporoensis* through interspecific mating is particularly important; it leads to the production of inviable hybrids. Consequently, queens that engage in interspecific mating, especially if they are monandrous, cannot establish viable colonies with workers (Kanbe et al. 2008; Kondo et al. 2009). *Bombus terrestris* is reportedly a monandrous species in its native range in Europe (Estoup et al. 1995; Schmid-Hempel and Schmid-Hempel 2000); the two subspecies, *Bombus hypocrita hypocrita* and *B. hypocrita sapporoensis*, were initially considered monandrous in Japan (Kinota et al. 2013).

In contrast, Inoue et al. (2012) reported that the queens of *B. terrestris* in Hokkaido exhibit polyandry, as determined through genetic markers applied to individuals collected in the field. Surprisingly, minimal attention has been directed toward exploring the relationship between reproductive interference and polyandry. Findings by Inoue et al. (2012) suggest that the mating frequency of queens increases in response to the rising frequencies of interspecific mating with the native bumblebee, *B. hypocrita sapporoensis*, potentially as a strategy to mitigate reproductive interference (Tsuchida et al. 2019).

Interspecific mating between invasive *B. terrestris* and native *B. hypocrita sapporoensis* has been observed in the wild, resulting in inviable eggs (Kanbe et al. 2008; Kondo et al. 2009). This reproductive interference has the potential to cause species exclusion (Ribeiro and Spielman 1986; Kuno 1992; Liu et al. 2007; Gröning and Hochkirch 2008; Kishi et al. 2009; Crowder et al. 2010), and may contribute to species extinction in the sympatric area. Conversely, reproductive interference can foster coexistence and result in parapatry between two interacting species (Ribeiro and Spielman 1986; Kuno 1992). However, there is an additional previously overlooked possibility—if there are variations in queen mating frequency within a population, reproductive interference could have more detrimental impacts on monandrous queens than on polyandrous queens. Monandrous queens may be unable to produce viable offspring workers if they exclusively copulate with males of another species. If this hypothesis is correct, polyandrous queens could be rapidly selected over successive generations. Moreover, we expected native queens to mate more frequently in areas where the chance of interspecific mating was more prevalent due to the higher abundance of invasive species. Briefly, our hypothesis suggests that reproductive interference selects for polyandry in queens, and (2) its effects are more pronounced in regions with a high abundance of partner species engaging in reproductive interference.

Therefore, we estimated the mating frequencies of queens of *B. terrestris* and *B. hypocrita sapporoensis*. As a reference species, we examined another Japanese bumblebee, *B. hypocrita hypocrita*, which is allopatrically distributed with *B. terrestris* in Japan. We assessed the levels of polyandry of the three species using genetic markers, eliminating the possibility of worker drift as a confounding factor.

## Materials and methods

### Insects and collection sites

**Fig. 1:**
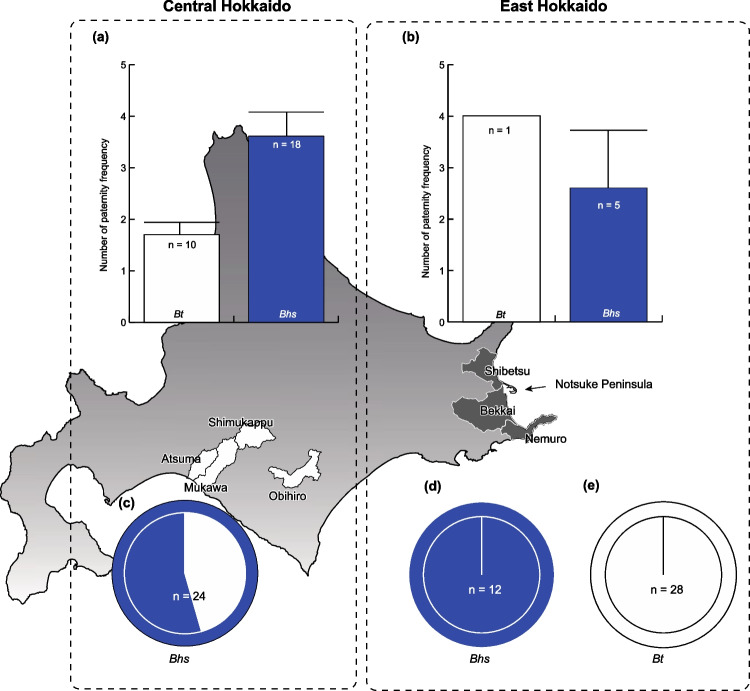
**a** The numbers of mating partners for queens (bar plots) of *Bombus terrestris* (*Bt*) are shown in white; for *Bombus hypocrita sapporoensis* (*Bhs*), the numbers are shown in blue. The estimated number for *Bt* in Central Hokkaido was obtained from Inoue et al. (2012) with a few modifications (see text), who collected samples from Atsuma and Mukawa Towns in Central Hokkaido. In this study, we collected samples from our rearing colonies derived from queens collected in Central Hokkaido (Shimukappu Village and Obihiro City). **b** Similarly, we estimated these numbers for *Bhs* from the rearing colonies derived from queens collected in East Hokkaido (Shibetsu Town, Bekkai Town, and Nemuro City). Each bar represents SE. We did not estimate these numbers for *Bt* in East Hokkaido. **c**, **d**, **e** The results of spermatozoa genotyping of *Bhs* and *Bt* queens. Each outer circle represents the spermatheca of either *Bhs* (blue) or *Bt* (white) queens. Pie charts within the inner circles indicate the paternity frequencies for either *Bhs* (blue) or *Bt* (white), as detected by our genotyping analyses. The paternity frequency for *Bhs* in central Hokkaido was obtained from Kondo et al. (2009). Small islands belonging to Hokkaido are excluded from this figure

We analyzed bumblebees collected in three distinct areas in this study: East Hokkaido (northernmost island of Japan), Central Hokkaido, and Central Honshu (main island of Japan). In Central Hokkaido, we collected samples using insect nets from Obihiro City and Shimukappu Village (Fig. 1), where a significant influx of *B. terrestris* (hereafter, *Bt*) queens was observed beginning around 2003 (Inoue, personal observation). Central Hokkaido contains a hilly landscape, farmland dedicated to vegetable production, and a ski resort. The ski slope area is rich in dandelions from spring to summer, providing abundant honey and pollen for bumblebees. On the other hand, East Hokkaido is a dairy region and has a flatter terrain than Central Hokkaido. In East Hokkaido, we collected *B. hypocrita sapporoensis* (hereafter, *Bhs*) samples from the Notsuke Peninsula of Shibetsu Town, the Nosappu Peninsula of Nemuro City, and Bekkai Town, where the invasion of *Bt* was first reported in 2007 (Inoue et al. 2009). Most areas of the Notsuke Peninsula are located within Notsuke-Furen Natural Park, officially designated for protecting natural fauna and flora. Central Hokkaido represents a region characterized by mass invasions of *Bt*. In contrast, East Hokkaido serves as the front line of the invasion (Fig. 1). Conversely, *Bt* has not been noticeably naturalized in Honshu; the native subspecies *B. hypocrita hypocrita* (hereafter, *Bhh*) is prevalent. We collected *Bhh* samples in Honshu. Hokkaido and Honshu are separated by the biogeographical barrier known as the Blakiston Line, and the subspecies *Bhs* is distributed in Hokkaido.

### Spermatheca inspection

Queens of both *Bt* and *Bhs* (*n* = 58 and 116, respectively) were collected from Notsuke Peninsula, excluding the Notsuke-Furen Natural Park in East Hokkaido during early June 2011 and 2012. These specimens were stored at – 20 °C until dissection for spermatheca collection.

### Rearing experiments

We collected queens of *Bhh* from fields around Ibi County in Gifu Prefecture and Minami-Azumi County of Nagano Prefecture on Honshu from mid-April to the end of May 2003 (*n* = 9) using insect nets. Additionally, queens of *Bhs* were collected in Central Hokkaido (Sinukappu Village and Obihiro City) and East Hokkaido (Shibetsu Town, Bekkai Town, and Nemuro City) from mid-April to the end of May 2010 and 2011 (*n* = 234). Importantly, queens of *Bhs* were not collected within the confines of the Notsuke-Furen Natural Park area.

The queens of the two subspecies were released into individual small wooden boxes (10.0 × 18.0 × 8.5 cm) that had been divided into two compartments using a partition plate (smaller container: 10.0 × 8.0 × 8.5 cm; larger container: 10.0 × 10.0 × 8.5 cm), allowing each adult to move freely between the compartments through a hole (2.0 × 2.0 cm). Each box was placed in the dark at 23–26 °C and 60–90% relative humidity. A pollen ball made from pollen powder mixed with 50% sucrose solution and a cotton ball soaked in 50% sucrose solution were placed in each box using the caps of plastic drinking water bottles (e.g., Kanbe et al. 2008). To induce oviposition by each queen, we installed a paper sheet on which a pupa of *Bombus ignites* had been attached with glue.

After confirmation of the queen’s oviposition, each colony was transferred to a large wooden box (20 × 30 × 15 cm) that had been divided into two compartments using a partition plate (smaller container: 20.0 × 11.7 × 15.0 cm; larger container: 20.0 × 18.4 × 15.0 cm), allowing each adult to move freely between the compartments through a hole (2.0 × 2.0 cm). An ample amount of pollen ball and 50% sucrose solution were supplied once every 2 days. For *Bhh*, we induced 15 queens to establish colonies, and 12 of the 15 queens successfully produced reproductive offspring. For *Bhs*, 48 of the 234 queens successfully produced reproductive offspring.

### Extraction and sequencing of DNA from spermatheca

We extracted DNA from spermatheca of the queens collected in East Hokkaido in accordance with the method of Peters et al. (1995). We dissected queens of *Bhs* (*n* = 116) and *Bt* (*n* = 58) and collected each spermatheca. For the extracted DNA, rhodopsin gene sequences were analyzed using an Applied Biosystems 3730 Genetic Analyzer (Applied Biosystems, Foster City, CA, USA). Detailed procedures are provided in the Supplementary Information.

### Colony genetic structure

We collected samples from the breeding colonies in our laboratory. We genotyped 1 queen, 10 arbitrarily selected workers, and 24–80 males from 8 colonies of *Bhh* collected in 2010, using 5 primer pairs developed by Estoup et al. (1995, 1996). We genotyped 1 queen, 8 arbitrarily selected workers, and 15–23 males from 5 colonies of *Bhs* collected in 2010, using 5 primer pairs developed by Estoup et al. (1995, 1996). We also genotyped a queen, 10 arbitrarily selected workers, and 15 males from 18 colonies of *Bhs* collected in 2012, using 5 primer pairs developed by Stolle et al. (2009). Each PCR product was analyzed as described in the Supplementary Information.

### Estimations of paternity frequency and worker relatedness

We inferred the paternity frequencies of the queen and worker relatedness for 18 colonies of *Bhs* and 6 colonies for *Bhh*. Because bumblebees are haplodiploid, we assumed that the male partner’s genes were genes shared by the workers but not the queen. From the inferred pedigrees, we determined the number of mating partners of the queen and calculated three measures of effective paternity frequency, considering differences in sample size and paternity unevenness: (1) ${m}_{e}=1/(\sum {q}_{i}^{2})$(Starr 1984), (2) ${m}_{ep}=(n-1)/(n\sum {q}_{i}^{2}-1)$ (Pamilo 1993), where *n* is the sample size, and (3) ${m}_{ep2}={(n-1)}^{2}/\left[\sum {q}_{i}^{2}\left(n+1\right)\left(n-2\right)+3-n\right]$(Nielsen et al. 2003).

Genetic relatedness among workers (*G*) was calculated using the following equation: $G=1/4+[0.5(1/{m}_{e})]$ (Pamilo 1993).

We estimated the frequencies of males derived from workers using the equation described by Arévalo et al. (1998):

$$L=K\prod_{j=1}^{{N}_{m}}\left(Q\prod_{j=1}^{n}{f}_{\left(q\right)i,j}+(1-Q)\prod_{j=1}^{n}{f}_{\left(w\right)i,j}\right)$$

where *Q* is the frequency of males derived from the queen; (*1* − *Q*) is the frequency of males derived from workers; *L* is the likelihood; *N*_*m*_ is the number of males; *n* is the number of loci; and *f*_*(q)i,j*_, and *f*_*(w)i,j*_ are the frequencies of male alleles considered in the queen and the workers for the *i*th male at the *j*th locus, respectively. We obtained the maximum likelihood estimator of *Q* when* L* reached the largest value.

Non-detection error (*d*_*p*_) is the probability that two males within a population in Hardy–Weinberg equilibrium have identical genotypes at all loci as follows:

$${d}_{p} = \prod \left(\sum {p}_{i}^{2}\right)j$$

where *p*_*i*_ denotes the allele frequency at each locus *j* (Boomsma and Ratnieks 1996).

Data previously reported by Inoue et al. (2012) were slightly modified and integrated with our own data to illustrate the numbers of mating partners for queens in Central and Eastern Hokkaido (Fig. 1). The modifications were outlined as follows: we compiled the data from Inoue et al. (2012) and categorized them into the two respective regions. Specifically, the data from Inoue et al. (2012) encompassed 22 colonies in Central Hokkaido and one colony in East Hokkaido (BtN-1 in Inoue et al. 2012), each designated within separate regions. Subsequently, we excluded data for seven *Bt* colonies lacking original queens in their data set due to incomplete elimination of the possibility of worker drift.

### Density estimation in the field

We estimated the relative densities of each *Bombus* species in Central (Mukawa Town) and East Hokkaido (Notsuke Peninsula) in accordance with the method of Inoue et al. (2008, 2009). The abundance of bumblebee species was surveyed in the monitoring area in June and August of 2011 and 2012. Through censuses conducted along roads, riverbeds, and farmland ridges, we recorded the date, time, location, habitat types, bumblebee species, caste (queen, worker, or male), and plant species with flowers visited. We captured as many individuals of *Bhs*, *Bt*, and other native species as possible. We summed the numbers of castes to determine the total number for each species, then calculated the relative densities of *Bt* and *Bhs* among all bumblebees caught in each area and the Shannon–Wiener index from these censuses.

All statistical analyses were conducted using R 4.3.2.

## Results

In accordance with the method of Inoue et al. (2012), we inferred the genotype of the queen’s mating partners in each colony from the genotypes of the queen and workers (Table S1). Based on these data, we estimated the numbers of mating partners. In Central Hokkaido, the mean number of mating partners for *Bt* queens was 1.70 ± 0.24 SE (*n* = 10), and 50% (5/10) of the queens were polyandrous. Similarly, the mean number of mating partners for *Bhs* queens was 3.61 ± 0.47 (*n* = 18, from Bhs_01 to Bhs_20), and 88.9% (16/18) of the queens were polyandrous. In East Hokkaido, the mean number of mating partners for *Bhs* queens was 2.60 ± 1.12 (*n* = 5, from Bhs_A to Bhs_E), and 60% (3/5) of the queens were polyandrous. For *Bt*, the mean number of mating partners was 4 (1/1). These numbers for *Bhs* in Central Hokkaido did not significantly differ from the numbers in East Hokkaido (*t*_*cal*_ = 0.985, *P* = 0.362). Although all *Bhh* queens were monandrous (Table S2), most *Bhs* queens were polyandrous. We found no evidence of interspecific mating between *Bhs* and *Bt* in East Hokkaido (Fig. 1, Table S3).

We estimated the proportions of workers sired by *i*th males for 23 *Bhs* colonies and 8 *Bhh* colonies (Table S2). The mean estimated percentages of males derived from workers were 51.5% ± 7.18% for *Bhs* and 13.9% ± 9.7% for *Bhh*.

The relationship between the number of mating partners for the queen and the frequencies of worker-derived males (%) for 23 colonies of *Bhs* (Fig. S1) was not statistically significant (*P* = 0.315), indicating that workers reproduced independently of the queen’s mating frequency.

We summarized the bumblebee density estimates and species diversities estimated by Shannon–Wiener indices for this study and previous reports in East and Central Hokkaido (Table 1). The results indicated that the density of *Bhs* did not differ between the two regions, although the density of *Bt* was significantly higher in Central Hokkaido than in East Hokkaido. Conversely, the diversity index was significantly higher in East Hokkaido than in Central Hokkaido.

**Table 1:** Densities (bumblebees/person/h) of all bumblebee species, *Bombus terrestris* (*Bt*), and *B. hypocrita sapporoensis* (*Bhs*), and Shannon–Wiener index in Central Hokkaido and East Hokkaido

	Year	Month	Density of all bumblebees	Density of *Bt*	Density of *Bhs*	Shannon–Wiener index	Reference
Central Hokkaido	2003	May–Sep	7.6	4	1.8	1.28	Inoue et al. (2008)
	2004	May–Sep	10.5	7.2	0.8	0.95	Inoue et al. (2008)
	2005	May–Sep	10.3	7.7	0.8	0.86	Inoue et al. (2008)
	2011	Jun	22.1	12.3	9.5	0.74	This study
Mean	–––	–––	**12.7**^**a**^	**7.8**^**b**^	**3.2**^**c**^	**0.96**^**d**^	
East Hokkaido	2007	Jun, Aug	10.3	0.3	3.4	1.55	Inoue et al. (2009)
	2011	Jun, Aug	23.7	1.4	1.9	1.33	This study
	2012	Jun, Aug	7.8	0.3	3.2	1.47	This study
Mean	–––	––-	**13.9**^**a**^	**0.7**^**c**^	**2.8**^**c**^	**1.45**^**e**^	

The comparisons of densities between Central and East Hokkaido for all bumblebees, *Bt*, and *Bhs* with different letters were found to be significantly different (*P* < 0.05) using the Welch test. Additionally, the comparison between the Shannon–Wiener index in Central and East Hokkaido was also significantly different using the Welch test (*P* = 0.017). The comparisons between the density of *Bt* and *Bhs* in Central and East Hokkaido with different letters were significantly different (*P* < 0.05) using the paired-*t* test

## Discussion

In this study, we predicted that *Bt* spillover would be more active in Central Hokkaido than in East Hokkaido, and that East Hokkaido would be an invasive front. Based on these predicted trends, we expected native queens to more frequently mate in Central Hokkaido, where spillover was more prevalent, than in areas with invasive fronts in East Hokkaido. To test these predictions, we estimated the mating frequencies of native *Bhs* queens and *Bhh* queens. Subsequently, we compared the mating frequencies of *Bhs* queens in Central and East Hokkaido. The results of the present study supported the first prediction: the density of *Bt* and the Shannon–Wiener diversity index was higher in Central Hokkaido than in East Hokkaido (Table 1). Recently, Inoue and Suzuki-Ohno (2023) reported similar results, noting that *Bt* populations have not increased on the Notsuke Peninsula based on continuous surveys from 2007 to 2019. They suggested that *Bt* may not be adapting to the cooler climate in this region. Another hypothesis is the narrowness of this peninsula. The Notsuke peninsula is a sand split, characterized by low elevation and narrow width due to the deposition of drift sand carried by coastal currents, probably making it a difficult terrain for new species to invade.

For the second aim, we modified the previous data. Because we wanted to compare mating frequencies after excluding the possibility of worker drift between colonies, we organized the results of Inoue et al. (2012) to fit this criterion. Indeed, the presence of drifting workers cannot be ignored and can be considered a worker reproductive strategy, rather than an exceptional phenomenon (Blacher et al. 2013; Zanette et al. 2014). Our modifications changed the original numbers for *Bt* (2.72 ± 0.55, *n* = 18) to 1.70 ± 0.24 (*n* = 10) in Central Hokkaido and 4 (*n* = 1) in East Hokkaido (Nemuro City). Despite such changes, the present data did not alter the overall results of Inoue et al. (2012), which indicated that queens of *Bt* were polyandrous in Central Hokkaido. Moreover, we found that the queens of *Bh*s were polyandrous in both Central and East Hokkaido (Fig. 1). There is evidence that *Bt* queens are monandrous in Europe where they are native (Estoup et al. 1995; Schmid-Hempel and Schmid-Hempel 2000). Our analyses showed that the queens of native *Bhh*, a subspecies of *Bhs*, were distributed allopatrically on Honshu; all colony queens were monandrous.

It is unclear how often *Bhs* queens mated before the *Bt* spillover occurred in this region. Previously, Kinota et al. (2013) noted that *Bhs* may primarily be monandrous, but such a tendency has not been confirmed by genetic studies. However, because the queens of the allopatric subspecies *Bhh* were monandrous in this study and many *Bombus* species are generally monandrous, it is reasonable to infer that *Bhs* was monandrous prior to the encounter with *Bt* in Hokkaido. In many social insects, queen monandry is considered the ancestral trait and polyandry is a derived trait; considering that many species in the genus *Bombus* (in stricto) are primarily monandrous, the polyandry we observed in *Bhs* was also regarded as a derived trait. We have summarized the frequencies of queen mating for *Bombus* species at the subgenus level in Table S4. Although the number of colonies surveyed seems insufficient, in *Pyrobombus*, several species (e.g., *Bombus hypnorum*) are polyandrous. However, the effective mating frequency of most species is very close to 1, with a few exceptions in which sample size has been insufficient. From these lines of evidence and our results, it could be safe to conclude that *Bt* and *Bhs* queens shifted from monandry to polyandry during the spread of *Bt* to Hokkaido. However, to confirm this, future studies on the mating frequency of *Bhs* on isolated islands belonging to Hokkaido not invaded by *Bt* are needed.

Interspecific mating between invasive *Bt* and native *Bhs* has been observed in the wild, resulting in inviable eggs (Kanbe et al. 2008; Kondo et al. 2009), a component of reproductive interference (Tsuchida et al. 2019). The frequency of interspecific mating was 0.302 among *Bhs* queens mainly collected in Central Hokkaido. Such reproductive interference via interspecific mating has the potential to lead to species exclusion (Ribeiro and Spielman 1986; Kuno 1992; Liu et al. 2007; Gröning and Hochkirch 2008; Kishi et al. 2009; Crowder et al. 2010) and may contribute to species extinction from the sympatric area. Conversely, reproductive interference can foster coexistence and result in parapatry between two interacting species (Ribeiro and Spielman 1986; Kuno 1992). Additionally, we assumed that reproductive interference would have more detrimental impacts on monandrous queens than on polyandrous queens. Consequently, polyandrous queens could undergo rapid favorable selection over successive generations. The results presented here support these trends, whereby invasive *Bt* and its native counterpart *Bhs* became polyandrous. Reproductive interference is a potential driver of the evolution of polyandry in the wild (Tsuchida et al. 2019).

We detected no interspecific mating between *Bhs* and *Bt* in East Hokkaido. Kubo et al. (2023) recently reported that *Bhs* queens collected in Nemuro Peninsula, which corresponds to the region regarded as East Hokkaido in the present study, showed an interspecific mating frequency of 0.044. Although this frequency is low relative to the frequency in Central Hokkaido, their results indicated that interspecific mating also occurs in the East. Polyandrous *Bhs* queens were detected even in the invasion front of East, where interspecific mating was not detected in the present study. This result suggests that the multiple mating trait rapidly evolved since the introduction of *Bt* and spread to *Bt* and *Bhs* queens. Takeuchi et al. (2018) reported that no significant genetic population structure of *Bhs* was present in Hokkaido, suggesting that there are no apparent genetic barriers within Hokkaido. Such a population structure would allow relatively rapid dispersal of polyandrous traits.

In the present study, we showed that *Bt* and *Bhs* queens were polyandrous where their ecological niches overlapped and *Bt* spillover occurred. Reproductive interference may have been a driving force for this phenomenon. Of course, other possible causes may explain the observed polyandry in *Bhs*. For example, another native species, *Bombus florilegus*, is distributed in East Hokkaido and produces diploid males due to matched mating (Takahashi et al. 2008b). Polyandry may also be promoted to avoid the detrimental effects of such matched mating. Polyandry and reproductive interference are costly for the queen; thus, species identification between *Bt* and *Bhs* upon mating may be selected for in the future, and they may return to monandry. Follow-up studies will allow us to predict whether *Bt* and *Bhs* can coexist or whether *Bhs* will be eradicated through interspecific mating.

### Supplementary Information

Below is the link to the electronic supplementary material.

Supplementary file1 (DOCX 29 KB)

Supplementary file2 (DOCX 76 KB)

## Data Availability

The datasets generated during and/or analyzed during the current study are available from the corresponding author on reasonable request.
