# Supplementary material for: Polyandry may mitigate the negative impact of reproductive interference among bumblebees in Japan
Source: Naturwissenschaften. 2024 May 23;111(3):31. doi: 10.1007/s00114-024-01917-5 (PMC11116251; doi:10.1007/s00114-024-01917-5)
Supplement: Supplementary file 1 — Supplementary file1 (DOCX 29 KB) [file 114_2024_1917_MOESM1_ESM.docx]

Supplementary information

Supplementary file 1

1. **DNA extraction, Rhodopsin gene sequencing and Microsatellite Genotyping**

**DNA extraction**

For genotypic analyses of adults, DNA was extracted from a leg. The leg was immersed in liquid nitrogen within a 1.5 mL tube and smashed with a pestle. Subsequently, it was mixed with 50 µL of extraction buffer containing 150 mM NaCl, 10 mM Tris-HCl (pH 8.0), 1.0 mM EDTA, 10 µg of proteinase K, and 40 µg of Chelex 100 (Bio-Rad Laboratories). The tubes were incubated at 56°C for 2 h and at 99.9°C for 3 min ([Walsh *et al.* 1991](#_ENREF_72)). Each solution was then precipitated with ethanol and stored in TE buffer containing 10 mM Tris-HCl (pH8.0) and 1mM EDTA (pH8.0) at 4°C.

**Rhodopsin gene sequencing**

For Rhodopsin gene sequencing, DNA was extracted from spermatheca of queens collected in East Hokkaido following to the method outlined by Peters et al. (1995). No queens collected in Central Hokkaido and Honshu were inspected. A droplet of 50 μL of distilled water (DW) and 10% NaCl was placed on a glass slide with two dimples. Queens of *Bhs* (n = 116) and *Bt* (n = 58) were dissected, and each spermatheca was placed in the DW. Adhering tissues were removed, and the spermatheca was washed in DW. The washed spermatheca was then placed in the droplet of 10% NaCl, and the surface tissue was removed using a fine insect pin. When filled with sperm (Duvoisin et al. 1999) and covered with a fine membrane, the resulting opaque white sphere was gently torn, and the membrane was removed with fine forceps. The resulting sperm were expelled from the receptaculum seminis membrane (Duvoisin et al. 1999), collected using micro-pipet, and transferred into a 200 μL tube with 20 μL of 50 mM DTT and 20 μL of 500 mM KOH, then vortexed for 30 s. Subsequently, the tube was placed in a PCR thermal cycler (TAKARA TP600) at 65 °C for 10 min for deproteinization. The resulting solution was neutralized with 20 μL of 500 mM HCl and 6.66 μL of 500 mM Tris-HCl (pH 9.0) and was used for PCR reaction.

**Microsatellite genotyping**

In our experiments, we employed three sequential methods to determine the genoytpes using microsatellites.

1. *Bhh* collected in 2010:

We genotyped the extracted DNA using microsatellite markers (B11, B100, B124, B126, B132; Estoup et al. 1995, 1996). PCR was conducted with 1 µL of diluted genomic DNA (approximately 1ng) in a mixture consisting of 0.05 µL of forward primer (100 pmol/µL), 0.05 µL of reverse primer (100 pmol/µL), 1.2 µL of 10 mM dNTP mix, 0.08 µL of Taq polymerase (5 units/µL, TAKARA Ex Taq), 1.5 µL of 10 × buffer (provided with the polymerase, containing 1.5 mM MgCl_2_), and 11.62 µL of DW^2^ in a total volume of 15.5 µL. PCR was carried out using a thermal cycler (TAKARA TP600). Following denaturation for 5 min at 95 °C, the samples were subjected to 35 cycles of 30 s at 95 °C, 90 s at the annealing temperatures appropriate for the primer pair, and 30 s at 72°C, with a final extension for 30 min at 60 °C. The PCR products were electrophoresed on 8% polyacrylamide gels and visualized by silver staining ([Bassam *et al.* 1991](#_ENREF_4)). Genotyping was conducted using the Kodak 1D image analysis software. Genotype scoring and data entry were performed twice, and the scores were compared by M. I.

1. *Bhs* collected in 2011:

For *Bhs*, DNA extraction and genotyping were conducted using microsatellite markers (B11, B100, B124, B126, B132; Estoup et al. 1995, 1996) as previously described. PCR products were visualized using electrophoresis equipment (QIAxcel^TM^ Advanced System (Qiagen, Hilden, Germany).

1. *Bhs* collected in 2012:

DNA extracted from *Bhs* collected in 2012 was genotyped using six primer pairs (BTMS065, BTMS082, BTMS113, BTMS125, BTMS126, BTMS136) developed by Stolle et al. (2009). We used Type-it Microsatellite PCR Kit (Qiagen, N.V., Hilden , Germany) was employed for PCR. The forward primer of each marker was 5’-end-labeled with a fluorescent phosphoramidite (NED, 6-FAM, VIC, and PET). A primer mix was prepared, containing 1.25 µL of each primer mix containing each forward and reverse primer (100 pmol/ µL) and 340 µL of TE buffer (pH8.0), mixed with 6.25 µL of 2 × Type-it Multiplex PCR Master Mix, 4.5 µL of DW^2^, 0.5 µL of genomic DNA. After denaturation for 5 min at 95 °C, the sample underwent 32 cycles of 30 s at 95 °C, 90 s at 57 °C, and 30 s at 72°C, with a final extension for 30 min at 60 °C. PCR fragments were analyzed using 3500 Genetic Analyzer (Applied Biosystems^TM^, Bedford, USA) and Gene Mapper^®^ (Applied Biosystems^TM^, Bedford, USA) using an internal size-standard 500 LIZ. Additionally, for DNA extraction, each leg was immersed in liquid nitrogen within a 1.5 mL tube and pulverized with a pestle. Subsequently, it was mixed with 50 µL of extraction buffer containing 150 mM NaCl, 10 mM Tris-HCl (pH 8.0), 1.0 mM EDTA, 10 µg of proteinase K, and 40 µg of Chelex 100 (Bio-Rad Laboratories). The tubes were incubated at 56°C for 2 h and at 99.9°C for 3 min ([Walsh *et al.* 1991](#_ENREF_72)). Each solution was then precipitated with ethanol and maintained in TE buffer containing 10 mM Tris-HCl (pH8.0) and 1mM EDTA (pH8.0) at 4°C.
