# Supplementary material for: Polyandry may mitigate the negative impact of reproductive interference among bumblebees in Japan
Source: Naturwissenschaften. 2024 May 23;111(3):31. doi: 10.1007/s00114-024-01917-5 (PMC11116251; doi:10.1007/s00114-024-01917-5)
Supplement: Supplementary file 2 — Supplementary file2 (DOCX 76 KB) [file 114_2024_1917_MOESM2_ESM.docx]

Supplementary file 2:

**Table S1 Genotypes of queen (Q) and her mating partners, and the proportions of workers sired by *i*th males (*q_i_*) for each colony of *Bombus hypocrita sapporoensis*:** Queens of colony Bhs_A to C were collected at Notsuke peninsula, Bhs_D at Bekkai town and Bhs_E at Nosappu peninsula (Nemuro city) in East Hokkaido. In Central Hokkaido, queens of Bhs_01 to 12 were collected at Shimukappu town, and Bhs_13 at Obihiro city.

**Table S2 Estimated number of queen’s mating partner and mating frequencies for each queen, genetic relatedness among workers, and the frequencies of males derived by workers, for *Bombus hypocrita sapporoesis* (*Bhs*) and *B. hypocrita.hypocrita* (*Bhh*).**

**Table S3 Frequencies of interspecific mating between native *Bombus hypocrita sapporoensis* and invasive *B. terrestris* using rhodopsin gene sequences.**

**Table S4 Effective number of queen mating frequency for each bumblebee species. Figure S1** **The relationship between the number of paternity frequency and the frequency of worker-derived males (%)**. Open circles represent colonies in the Central Hokkaido, while shaded circles represent colonies in East Hokkaido. The relationship was not found to be significant (P = 0.315)

**Table S1 Genotypes of queen (Q) and her mating partners, and the proportions of workers sired by *i*th males (*q_i_*) for each colony of *Bombus hypocrita sapporoensis*:** Queens of colony Bhs_A to C were collected at Notsuke peninsula, Bhs_D at Bekkai town and Bhs_E at Nosappu peninsula (Nemuro city) in East Hokkaido. In Central Hokkaido, queens of Bhs_01 to 12 were collected at Shimukappu town, and Bhs_13 at Obihiro city.

|  |  | Genotypes (bp) of queen (Q) and her mating partners (m) | | | | | |  |
| --- | --- | --- | --- | --- | --- | --- | --- | --- |
| Colony | Caste | B11 | B96 | B100 | B119 | B121 |  | *q_i_* |
| Bhs_A | Q | 177/183 | 240/240 | 132/138 | 162/166 | 163/163 |  |  |
|  | m1 | 183 | 240 | 138 | 166 | 163 |  | 0.750 |
|  | m2 | 177 | 240 | 138 | 166 | 163 |  | 0.250 |
| Bhs_B | Q | 173/175 | 236/240 | 132/132 | 158/176 | 163/163 |  |  |
|  | m1 | 187 | 236(240) | 130 | 172 | 163 |  | 0.125 |
|  | m2 | 187 | 238 | 130 | 172 | 163 |  | 0.250 |
|  | m3 | 187 | 238 | 130 | 176 | 163 |  | 0.125 |
|  | m4 | 173 | 238 | 132 | 176 | 163 |  | 0.125 |
|  | m5 | 173(175) | 238 | 130 | 172 | 163 |  | 0.125 |
|  | m6 | 175 | 240 | 132 | 172 | 163 |  | 0.125 |
|  | m7 | 173(175) | 238 | 130 | 176 | 163 |  | 0.125 |
| Bhs_C | Q | 175/177 | 238/240 | 130/132 | 176/176 | 163/163 |  |  |
|  | m1 | 173 | 240 | 132 | 174 | 163 |  | 1 |
| Bhs_D | Q | 173/177 | 238/240 | 132/132 | 174/176 | 163/163 |  |  |
|  | m1 | 181 | 238 | 130 | 166 | 163 |  | 0.5 |
|  | m2 | 181 | 238(240) | 130 | 176 | 163 |  | 0.5 |
| Bhs_E | Q | 171/191 | 236/240 | 130/130 | 164/182 | 163/163 |  |  |
|  | m1 | 185 | 238 | 130 | 174 | 163 |  | 1 |
|  |  | Genotypes (bp) of queen (Q) and her mating partners (m) | | | | | | |
| Colony | Caste | BTMS065 | BTMS082 | BTMS113 | BTMS125 | BTMS126 | BTMS136 | *q_i_* |
| Bhs01 | Q | 219/225 | 351/359 | 351/381 | 110/112 | 157/167 | 157/167 | ---- |
|  | m1 | 221 | 351(397) | 364 | 106 | 177 | 177 | 0.10 |
|  | m2 | 221 | 364 | 364 | 106 | 177 | 177 | 0.80 |
|  | m3 | 221 | 381 | 364 | 106 | 177 | 177 | 0.10 |
| Bhs02 | Q | 211/213 | 351/372 | 351/372 | 120/122 | 153/167 | 153/167 | ---- |
|  | m1 | 237 | 397 | 359 | 120(122) | 153(167) | 147 | 0.09 |
|  | m2 | 217 | 397 | 359 | 120(122) | 147 | 147 | 0.91 |
| Bhs03 | Q | 215/219 | 351/359 | 351/359 | 110/116 | 163/171 | 163/171 | ---- |
|  | m1 | 221 | 397 | 351(359) | 122 | 155 | 155 | 0.31 |
|  | m2 | 221 | 397 | 348 | 122 | 155 | 155 | 0.69 |
| Bhs06 | Q | 213/223 | 351/357 | 351/357 | 106/108 | 153/170 | 153/171 | ---- |
|  | m1 | 209 | 397 | 351(357) | 110 | 157 | 157 | 0.36 |
|  | m2 | 209 | 397 | 355 | 110 | 157 | 157 | 0.64 |
| Bhs07 | Q | 221/223 | 351/378 | 348/378 | 106/112 | 157/163 | 157/161 | ---- |
|  | m1 | 231 | 351(378) | 351 | 114 | 157(163) | 157(161) | 0.27 |
|  | m2 | 231 | 351(378) | 351 | 114 | 155 | 155 | 0.09 |
|  | m3 | 221(223) | 351(378) | 351 | 114 | 155 | 155 | 0.09 |
|  | m4 | 231 | 347 | 351 | 114 | 157(163) | 157(161) | 0.36 |
|  | m5 | 221(223) | 347 | 351 | 114 | 157(163) | 155 | 0.09 |
|  | m6 | 231 | 347 | 351 | 114 | 155 | 155 | 0.09 |
| Bhs08 | Q | 219/221 | 347/359 | 346/355 | 108/110 | 157/163 | 157/163 | ---- |
|  | m1 | 215 | 347(359) | 357 | 108(110) | 177 | 177 | 0.29 |
|  | m2 | 215 | 347(359) | 357 | 114 | 177 | 177 | 0.29 |
|  | m3 | 215 | 397 | 357 | 108(110) | 177 | 177 | 0.14 |
|  | m4 | 215 | 397 | 357 | 114 | 177 | 177 | 0.29 |
| Bhs09 | Q | 221/223 | 364/397 | 364/396 | 110/112 | 147/173 | 153/173 | ---- |
|  | m1 | 217 | 364(397) | 357 | 114 | 155 | 171 | 0.17 |
|  | m2 | 217 | 359 | 357 | 114 | 147(173) | 171 | 0.50 |
|  | m3 | 217 | 359 | 357 | 114 | 155 | 171 | 0.33 |
| Bhs10 | Q | 213/221 | 355/359 | 351/355 | 104/112 | 155/171 | 155/171 | ---- |
|  | m1 | 213(221) | 355(359) | 357 | 108 | 163 | 163 | 0.45 |
|  | m2 | 213(221) | 351 | 357 | 108 | 163 | 163 | 0.36 |
|  | m3 | 213(221) | 369 | 357 | 118 | 163 | 163 | 0.18 |
| Bhs11 | Q | 219/221 | 347/364 | 351/364 | 110/112 | 155/177 | 157/177 | ---- |
|  | m1 | 231 | 378 | 378 | 118 | 183 | 183 | 1.0 |
| Bhs12 | Q | 215/219 | 359/397 | 348/359 | 106/110 | 155/157 | 155/163 | ---- |
|  | m1 | 225 | 397 | 396 | 106 | 153 | 153 | 0.45 |
|  | m2 | 225 | 397 | 396 | 116 | 153 | 153 | 0.36 |
|  | m3 | 225 | 351 | 396 | 116 | 153 | 147 | 0.09 |
|  | m4 | 225 | 351 | 396 | 116 | 163 | ---- | 0.09 |
| Bhs13 | Q | 219/221 | 369/397 | 368/372 | 104/118 | 155/163 | 155/163 | ---- |
|  | m1 | 223 | 369(397) | 355 | 114 | 173 | 173 | 0.08 |
|  | m2 | 223 | 355 | 355 | 114 | 173 | 173 | 0.83 |
|  | m3 | 219(221) | 355 | 355 | 114 | 173 | 173 | 0.08 |
| Bhs14 | Q | ---- | 347/359 | ---- | 112/128 | 157/167 | 157/163 | ---- |
|  | m1 | ---- | 347(359) | ---- | 108 | 157(167) | 157(163) | 0.07 |
|  | m2 | ---- | 347(359) | ---- | 108 | 177 | 177 | 0.07 |
|  | m3 | ---- | 369 | ---- | 108 | 157(167) | 157(163) | 0.21 |
|  | m4 | ---- | 369 | ---- | 108 | 177 | 157(163) | 0.07 |
|  | m5 | ---- | 397 | ---- | 108 | 157(167) | 157(163) | 0.36 |
|  | m6 | ---- | 397 | ---- | 108 | 157(167) | 177 | 0.07 |
|  | m7 | ---- | 397 | ---- | 108 | 177 | 177 | 0.14 |
| Bhs15 | Q | 221/225 | 347/372 | 346/372 | 110/114 | 167/173 | 167/173 | ---- |
|  | m1 | 231 | 351 | 351 | 118 | 163 | 157 | 1.0 |
| Bhs16 | Q | 219/221 | 359/372 | 359/372 | 106/116 | 167/183 | 157/167 | ---- |
|  | m1 | 223 | 359(372) | 351 | 104 | 173 | ---- | 0.07 |
|  | m2 | 223 | 347 | 351 | 104 | 173 | 157(167) | 0.29 |
|  | m3 | 223 | 347 | 351 | 104 | 173 | 183 | 0.07 |
|  | m4 | 223 | 347 | 351 | 104 | 173 | 171 | 0.21 |
|  | m5 | 223 | 347 | 351 | 104 | 157 | 157(167) | 0.21 |
|  | m6 | 223 | 347 | 351 | 104 | 157 | 183 | 0.07 |
|  | m7 | 223 | 347 | 351 | 104 | 157 | 171 | 0.07 |
| Bhs17 | Q | 219/231 | 351/397 | 351/396 | 112/122 | 163/167 | 163/171 | ---- |
|  | m1 | 221 | 359 | 359 | 112(122) | 163(167) | 157 | 0.10 |
|  | m2 | 221 | 359 | 359 | 106 | 163(167) | 157 | 0.90 |
| Bhs18 | Q | 209/223 | 364/372 | 368/372 | 110/112 | 167/183 | 157/183 | ---- |
|  | m1 | 209(223) | 364(372) | 368(372) | 114 | 171 | 167 | 0.21 |
|  | m2 | 209(223) | 397 | 368(372) | 114 | 171 | 167 | 0.07 |
|  | m3 | 209(223) | 397 | 364 | 114 | 171 | 167 | 0.36 |
|  | m4 | 221 | 397 | 368(372) | 114 | 171 | 167 | 0.07 |
|  | m5 | 221 | 397 | 364 | 114 | 171 | 167 | 0.29 |
| Bhs19 | Q | 223/225 | 355/397 | 355/372 | 108/116 | 163/167 | 153/167 | ---- |
|  | m1 | 231 | 355(397) | 355(372) | 110 | 157 | 157 | 0.21 |
|  | m2 | 223(225) | 372 | ---- | 110 | 163(167) | 157 | 0.07 |
|  | m3 | 231 | 372 | 355(372) | 110 | 163(167) | 157 | 0.21 |
|  | m4 | 231 | 372 | 368 | 110 | 163(167) | 157 | 0.21 |
|  | m5 | 231 | 372 | 355(372) | 108(116) | 163(167) | 157 | 0.07 |
|  | m6 | 231 | 372 | 355(372) | 110 | 157 | 157 | 0.14 |
|  | m7 | 231 | 372 | 368 | 110 | 157 | 157 | 0.07 |
| Bhs20 | Q | 221/223 | 355/397 | 351/359 | 110/114 | 147/167 | 157/167 | ---- |
|  | m1 | 231 | 355(397) | 396 | 120 | 155 | 157(167) | 0.36 |
|  | m2 | 231 | 355(397) | 396 | 120 | 155 | 177 | 0.09 |
|  | m3 | 231 | 364 | 396 | 120 | 155 | 157(167) | 0.55 |

**Table S2 Estimated number of queen’s mating partner and mating frequencies for each queen, genetic relatedness among workers, and the frequencies of males derived by workers, for *Bombus hypocrita sapporoesis* (*Bhs*) and *B. hypocrita.hypocrita* (*Bhh*)**.

| Colony | No. of workers analyzed | No. of queen’s mating partner | *m_e_* | *m_ep_* | *m_ep_*_2_ | Relatedness (G) | Males derived by workers (%) |
| --- | --- | --- | --- | --- | --- | --- | --- |
| Bhs_A | 8 | 2 | 1.6 | 1.75 | 1.70 | 0.56 | 60.2 |
| Bhs_B | 8 | 7 | 6.4 | 64 | 16 | 0.33 | 79.0 |
| Bhs_C | 8 | 1 | 1 | 1 | 1 | 0.75 | 89.8 |
| Bhs_D | 8 | 2 | 2 | 2.33 | 2.28 | 0.5 | 93.6 |
| Bhs_E | 8 | 1 | 1 | 1 | 1 | 0.75 | 14.8 |
| Mean ± SE | 8 | 2.60 ± 1.12 | 2.4 | 14.01 | 4.40 | 0.58 | 67.5 ± 11.8 |
| Bhs_01 | 10 | 3 | 1.52 | 1.61 | 1.59 | 0.58 | 9.3 |
| Bhs_02 | 11 | 2 | 1.20 | 1.22 | 1.21 | 0.67 | 50.0 |
| Bhs_03 | 14 | 2 | 1.74 | 1.85 | 1.84 | 0.54 | 20.5 |
| Bhs_06 | 12 | 2 | 1.86 | 2.01 | 1.98 | 0.52 | 78.5 |
| Bhs_07 | 12 | 6 | 4.17 | 6.05 | 5.62 | 0.37 | 48.7 |
| Bhs_08 | 14 | 4 | 3.77 | 4.63 | 4.45 | 0.38 | 94.1 |
| Bhs_09 | 8 | 3 | 2.57 | 3.33 | 3.07 | 0.44 | 70.8 |
| Bhs_10 | 12 | 3 | 2.69 | 3.26 | 3.15 | 0.44 | 50.1 |
| Bhs_11 | 10 | 1 | 1 | 1 | 1 | 0.75 | 27.0 |
| Bhs_12 | 12 | 4 | 2.81 | 3.46 | 3.34 | 0.43 | 91.5 |
| Bhs_13 | 12 | 3 | 1.41 | 1.48 | 1.47 | 0.60 | 61.2 |
| Bhs_14 | 14 | 7 | 4.67 | 6.56 | 6.19 | 0.36 | 41.5 |
| Bhs_15 | 14 | 1 | 1 | 1 | 1 | 0.75 | 0.0 |
| Bhs_16 | 14 | 7 | 5.16 | 7.71 | 7.18 | 0.35 | 92.8 |
| Bhs_17 | 10 | 2 | 1.22 | 1.25 | 1.24 | 0.66 | 16.7 |
| Bhs_18 | 15 | 5 | 3.77 | 4.64 | 4.49 | 0.38 | 46.2 |
| Bhs_19 | 14 | 7 | 5.76 | 9.76 | 8.90 | 0.34 | 55.6 |
| Bhs_20 | 11 | 3 | 2.28 | 2.60 | 2.53 | 0.47 | 91.9 |
| Mean ± SE | 12.2 ± 0.45 | 3.61 ± 0.47 | 2.70 ± 0.36 | 3.52 ± 0.60 | 3.35 ± 0.55 | 0.50 ± 0.03 | 51.5 ± 7.18 |
| Bhh_01 | 10 | 1 | 1 | 1 | 1 | 0.75 | 9.3 |
| Bhh_02 | 10 | 1 | 1 | 1 | 1 | 0.75 | 20.1 |
| Bhh_04 | 10 | 1 | 1 | 1 | 1 | 0.75 | 28.4 |
| Bhh_05 | 10 | 1 | 1 | 1 | 1 | 0.75 | 14.7 |
| Bhh_07 | 10 | 1 | 1 | 1 | 1 | 0.75 | 0.0 |
| Bhh_08 | 10 | 1 | 1 | 1 | 1 | 0.75 | 11.1 |
| Mean ± SE | 10 | 1 | 1 | 1 | 1 | 0.75 | 13.9 ± 9.7 |

$$m_{e}=1/(\Sigma q_{i}^{2})$$

$$m_{ep}=(n-1)/(n\Sigma q_{i}^{2}-1)$$

$$m_{ep2}=\left( n-1 \right)^{2}/[\Sigma q_{i}^{2}\left( n+1 \right)\left( n-2 \right)+3-n]$$

The queens of colonies Bhs_A to Bhs_D were collected at Notsuke Peninsula, and the queen of Bhs_E was collected at Nosappu Peninsula (Nemuro City) in East Hokkaido. The queens of colonies Bhs_01 to Bhs_12 were collected in Shimukappu Village, and those of Bhs_13 to Bhs_20 were collected in Obihiro City in Central Hokkaido. All Bhh colonies were collected in Ibi County of Gifu Prefecture and Minami-Azumi County of Nagano Prefecture on the Main island of Japan.

**Table S3 Frequencies of interspecific mating between native *Bombus hypocrita sapporoensis* and invasive *B. terrestris* using rhodopsin gene sequences.**

| Species | Year | No. of queens analyzed | No. of individuals with allospecific rhodopsin genes |
| --- | --- | --- | --- |
| *Bombus hypocrita sapporoensis* | 2011 | 22 | 0 |
|  | 2012 | 29 | 0 |
|  | total | 51 | 0 |
| *B. terrestris* | 2011 | 21 | 0 |
|  | 2012 | 12 | 0 |
|  | total | 33 | 0 |

**Table S4 Effective number of queen mating frequency for each bumblebee species.**

| Sub-geneus | Species name | No. of colonies | Effective number of mating# | Polyandrous queen/sampled queen | Reference |
| --- | --- | --- | --- | --- | --- |
| *Bombias* | *Bombus auricomus* | 1 | 1 |  | Payne *et al*. (2003) |
| *Bombus* | *B. affinis* | 1 | 1 |  | Payne *et al*. (2003) |
|  | *B. florilegus* | 14 | 1 |  | Takahashi *et al*. (2008a) |
|  | *B. hypocrita hypocrita* | 6 | 1 |  | This study |
|  | *B. h. sapporoensis* | 18 | 3.52 | 16/18 | This study (Central Hokkaido) |
|  | *B. h. sapporoensis* | 5 | 2.60 | 3/5 | This study (East Hokkaido) |
|  | *B. ignitus* | 7 | 1 |  | Takahashi *et al*. (2008b) |
|  | *B. lucorum* | 1 | 1 |  | Estoup *et al*. (1995b) |
|  |  | 12 | 1 |  | Schmid-Hemple & Schmid-Hemple (2000) |
|  | *B. terrestris* | 2 | 1 |  | Estoup *et al*. (1995b) |
|  |  | 17 | 1 |  | Schmid-Hemple & Schmid-Hemple (2000) |
|  |  | 10 | 1.70^$^ | 5/10 | Inoue *et al*. (2012) (Central Hokkaido) |
| *Fervidobombus* | *B. fervidus* | 1 | 1 |  | Payne *et al*. (2003) |
| *Megabombus* | *B. diversus* | 3 | 1 |  | Kokuvo *et al*. (2009) |
|  | *B. hortorum* | 5 | 1 |  | Schmid-Hemple & Schmid-Hemple (2000) |
| *Melanobombus* | *B. lapidarius* | 20 | 1 |  | Estoup *et al.* (1995b) |
|  |  | 11 | 1 |  | Schmid-Hemple & Schmid-Hemple (2000) |
|  | *B. sicheli* | 2 | 1 |  | Schmid-Hemple & Schmid-Hemple (2000) |
| *Pyrobombus* | *B. ardens* | 5 | 1 |  | Kokuvo *et al*. (2009) |
|  | *B. bimaculatus* | 4 | 1.06 | 1/4 | Payne *et al.* (2003) |
|  | *B. hypnorum* | 3 | ----- | 2/3 | Estoup *et al*. (1995b) |
|  |  | 17 | 1.12 | 4/17 | Schmid-Hemple & Schmid-Hemple (2000) |
|  |  | 14 | 1.26 | 7/14 | Paxton *et al*. (2001) |
|  |  | 10 | 1 |  | Brown *et al*. (2003) |
|  | *B. impatiens* | 10 | ----- | 2/10 | Cnaani *et al*. (2002) |
|  |  | 11 | 1.07 | 1/9 | Payne *et al*. (2003) |
|  | *B. mixtus* | 1 | 3.57 | 1/1 | Payne *et al*. (2003) |
|  | *B. pratorum* | 35 | 1 |  | Estoup *et al*. (1995b) |
|  |  | 5 | 1 |  | Schmid-Hemple & Schmid-Hemple (2000) |
|  | *B. ternarius* | 1 | 2.04 | 1/1 | Payne *et al*. (2003) |
|  | *B. vagans* | 4 | 1 |  | Payne *et al*. (2003) |
|  | *B. wilmattae* | 9 | 1.16 | 2/9 | Huth-Schwarz *et al*. (2001) |
| *Separatobombus* | *B. griseocollis* | 1 | 1 |  | Payne *et al*. (2003) |
| *Thoracobombus* | *B. honshuensis* | 1 | 1 |  | Kokuvo *et al*. (2009) |
|  | *B. pascuorum* | 6 | 1 |  | Schmid-Hemple & Schmid-Hemple (2000) |
| *Psithyrus** | *B. citrinus* | 2 | 2.13 | 2/2 | Payne *et al*. (2003) |
|  | *B. insularis* | 3 | 1 |  | Payne *et al*. (2003) |

: indicates a social parasite

#: represents the effective number of matings calculated using one of the methos by Starr (1984), Pamilo (1993), and Nielsen et al. (2003), all based on the harmonic mean.

$: the estimated number was re-calculated after removing a colony collected in East Hokkaido (Notseke Peninsula).
